# TransExION: a transformer based explainable similarity metric for comparing IONS in tandem mass spectrometry

**DOI:** 10.1186/s13321-024-00858-5

**Published:** 2024-05-28

**Authors:** Danh Bui-Thi, Youzhong Liu, Jennifer L. Lippens, Kris Laukens, Thomas De Vijlder

**Affiliations:** 1 Computer Science Department, University of Antwerp, Middelheimlaan 1, 2020 Antwerp, Belgium; 2 Therapeutic Development and Supply, Janssen Pharmaceutica N.V., Turnhoutseweg 30, 2340 Beerse, Belgium

**Keywords:** Tandem mass spectrometry, Small molecule identification, Spectral similarity, Structural similarity, Explainable deep neural network

## Abstract

**Abstract:**

Small molecule identification is a crucial task in analytical chemistry and life sciences. One of the most commonly used technologies to elucidate small molecule structures is mass spectrometry. Spectral library search of product ion spectra (MS/MS) is a popular strategy to identify or find structural analogues. This approach relies on the assumption that spectral similarity and structural similarity are correlated. However, popular spectral similarity measures, usually calculated based on identical fragment matches between the MS/MS spectra, do not always accurately reflect the structural similarity. In this study, we propose TransExION, a Transformer based Explainable similarity metric for IONS. TransExION detects related fragments between MS/MS spectra through their mass difference and uses these to estimate spectral similarity. These related fragments can be nearly identical, but can also share a substructure. TransExION also provides a post-hoc explanation of its estimation, which can be used to support scientists in evaluating the spectral library search results and thus in structure elucidation of unknown molecules. Our model has a Transformer based architecture and it is trained on the data derived from GNPS MS/MS libraries. The experimental results show that it improves existing spectral similarity measures in searching and interpreting structural analogues as well as in molecular networking.

**Scientific Contribution:**

We propose a transformer-based spectral similarity metrics that improves the comparison of small molecule tandem mass spectra. We provide a post hoc explanation that can serve as a good starting point for unknown spectra annotation based on database spectra.

**Supplementary Information:**

The online version contains supplementary material available at 10.1186/s13321-024-00858-5.

## Introduction

Tandem mass spectrometry (MS/MS) is a technique in which selected ions or precursor ions, obtained from chemical compounds, are fragmented into smaller product ions. The mass-to-charge ratio (*m/z)* and intensities of these product ions are recorded in a MS/MS spectrum, which reveals insights into the chemical structure of the precursor ion. MS/MS spectra are widely used for small molecule identification in modern analytical chemistry. Several MS/MS spectral libraries have been published, including GNPS [1], HMDB [2], METLIN [3], and MassBank [4].

Along with the growth of MS/MS libraries, computational methods for small molecule structure prediction from MS/MS spectra have emerged. In general, we can categorize these methods into three main approaches, namely spectral library search, structure database search, and database free approaches [5]. Spectral library searching compares query spectra against a spectral library based on a similarity measure, while structure database searching compares the query spectra against compounds in a structure database using intermediate representation. Different intermediate representation methods have been proposed for the latter, including transforming MS/MS spectra into molecular fingerprints [6–10], generating *in silico* MS/MS spectra from reference compounds [11–19], and matching spectra and reference compound embeddings [20]. The database free methods, such as MassGenie [21] and MSNovelist [5], require neither spectral libraries nor compound structure databases for structure prediction. Instead, they generate SMILES strings, a specified notation for describing compound structures, directly from an unknown spectrum using machine learning.

Spectral library searching is usually the first method considered for small molecule identification tasks. Its major limitation is that spectral libraries contain a finite amount of structures, making the identification of completely new structures challenging. On the other hand, searching for similar structures (structure analogues) that have one or more substructures in common with the query compound can offer a good starting point for structural annotation. Several spectral similarity measures dedicated to analogue searching have been proposed, including classical measures and machine learning based measures. Classical similarity measures, such as Cosine, Modified Cosine and Neutral Loss Matching [22], are computed based on identical matches of fragment ions and neutral losses. The MS/MS spectra of similar small molecules can however appear very different as minor functional group changes of a matched substructure can drastically affect the fragmentation behavior. Even MS/MS spectra of the same molecule can vary profoundly as fragmentation depends on several parameters such as the type of mass analyzer used, the applied collision gas and energy, etc [23]. The measures based on identical matches are of limited use in searching structural analogues. Therefore, recent studies have explored other information for MS/MS spectrum alignment, attempting to improve the correlation between the spectral similarity and structural similarity.

SIMILE [24] uses all *m*/*z* differences amongst a MS/MS spectrum pair to estimate spectral similarity. It starts by transforming the matrix of *m*/*z* difference counts into a substitution matrix using Laplacian Embedding. The substitution matrix allows spectral alignment thus spectral similarity estimation in a similar fashion as protein sequence alignment. Although SIMILE considers *m*/*z* difference for the alignment of a single spectrum pair, it does not measure the importance of such difference throughout the spectral library, which can be done using machine learning. In fact, SIMILE should be complemented with classical measures when searching a spectral library for compound identification.

Meanwhile, the fast growing AI frameworks and the expansion of public MS/MS spectral libraries available for model training have enabled machine learning-driven spectral similarity prediction. Spec2Vec [25], an unsupervised method, adapts the language model Word2Vec [26] to describe the co-occurrences of fragments across large spectral data-sets. Furthermore, several supervised deep learning approaches, such as DeepMass [23] and MS2DeepScore [27], estimate spectral similarity that can directly reflect the structural similarity. In their training phase, both approaches start by pairing reference spectra from a spectral library before feeding the pairs into a deep neural network to predict the underlying structure similarity. While DeepMass uses the concatenated vectors of *m/z*, intensity, and other features of the spectral pair as input of fully-connected neural network layers, MS2DeepScore adopts a Siamese architecture to learn the structure similarity directly from binned spectra pairs. The Siamese network encodes each spectrum of the pair separately before calculating the Cosine similarity of two embeddings. Although the machine learning based methods can improve spectral library search by outperforming the classical measures, MS2DeepScore has demonstrated a higher prediction accuracy and implement-ability, especially since no additional meta-data or library data is required. In fact, MS2DeepScore has been used as a key measure in MS2Query [28], a tool for finding both structural analogues and exact matches from large scale spectral libraries. However, one major limitation of MS2DeepScore is the discrimination between highly similar structural analogues (Tanimoto scores say 0.8–0.9) and a near-complete chemical match (Tanimoto scores ${>}\,0.9$). Moreover, none of existing machine learning based methods, to our knowledge, provide an explainability assessment on the important spectral features used for model output.

Here, we present TransExION, a Transformer based Explainable similarity metric for ions observed in tandem mass spectrometry. Our main goal is to improve spectral library searching, especially in finding structural analogues. Hence, we have designed and trained a supervised deep learning model to predict MS/MS spectral similarity that accurately reflects the structural similarity. TransExION adopts a Transformer architecture and offers a post hoc explanation feature.

The major novelty of TransExION is that, in addition to aligned product ions and neutral losses, the mass difference between query and reference fragments are also considered for spectral similarity scoring. Undoubtedly, using mass differences extends the scope of spectral alignment from exact substructure matching towards finding substructures with minor modifications. TransExION receives mass difference matrices as input and returns a spectral similarity score. The interpret-ability analysis of such model focuses on the importance of mass difference between each pair of product ions/neutral losses (one from each spectrum). We observe that the mass difference of paired (one from each spectrum) product ions/neutral losses can imply not only small substructure differences but can also be exploited to infer a potential structural relationship.

The spectral similarity prediction by TransExION, along with its post hoc explanation, can greatly enhance the structure elucidation of unknown analytes by experts. The experimental results demonstrate that TransExION outperforms existing methods in retrieving structure analogues from the spectral library. Furthermore, the post hoc algorithm unravels the substructure links between unknown product ions and reference spectra of retrieved analogues.

## Methods

### Model architecture

**Fig. 1:**
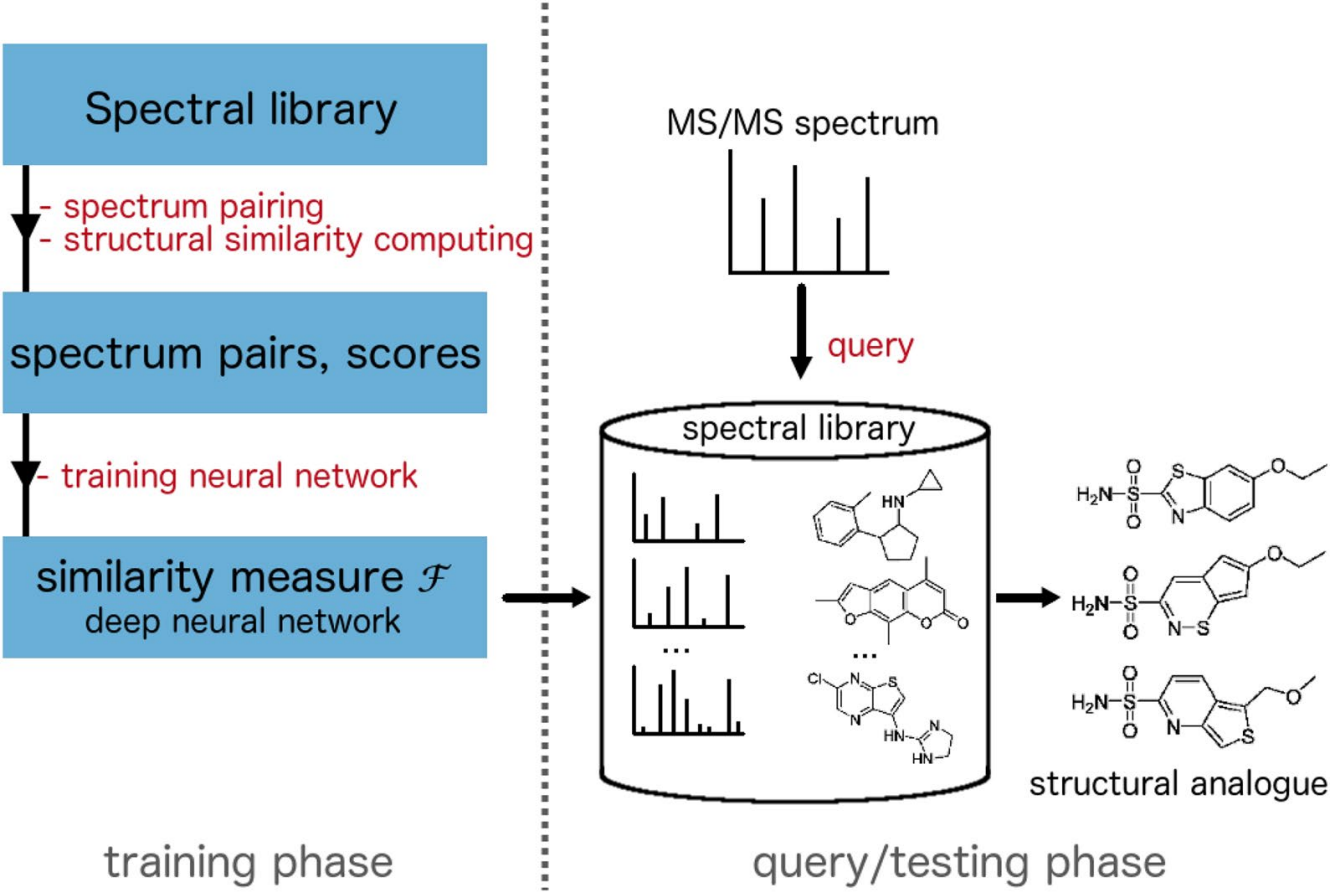
The training phase and query/testing phase of TransExION

We have built a deep neural network to estimate the spectral similarity between two MS/MS spectra, which can be used later as a proxy for structural similarity. Figure 1 presents an overview of the training phase and query/testing phase of our method. To create training data, we randomly sample pairs of spectra from a spectral library and calculate the corresponding structure similarity scores. These spectrum pairs and their structure similarity scores are used as the model input and output labels, respectively. During the testing phase, the query spectrum is paired with each reference spectrum in the spectral library so that the model can predict similarity scores for structure candidates one after another. Structural analogues are retrieved by selecting reference compounds with the highest predicted scores.

**Fig. 2:**
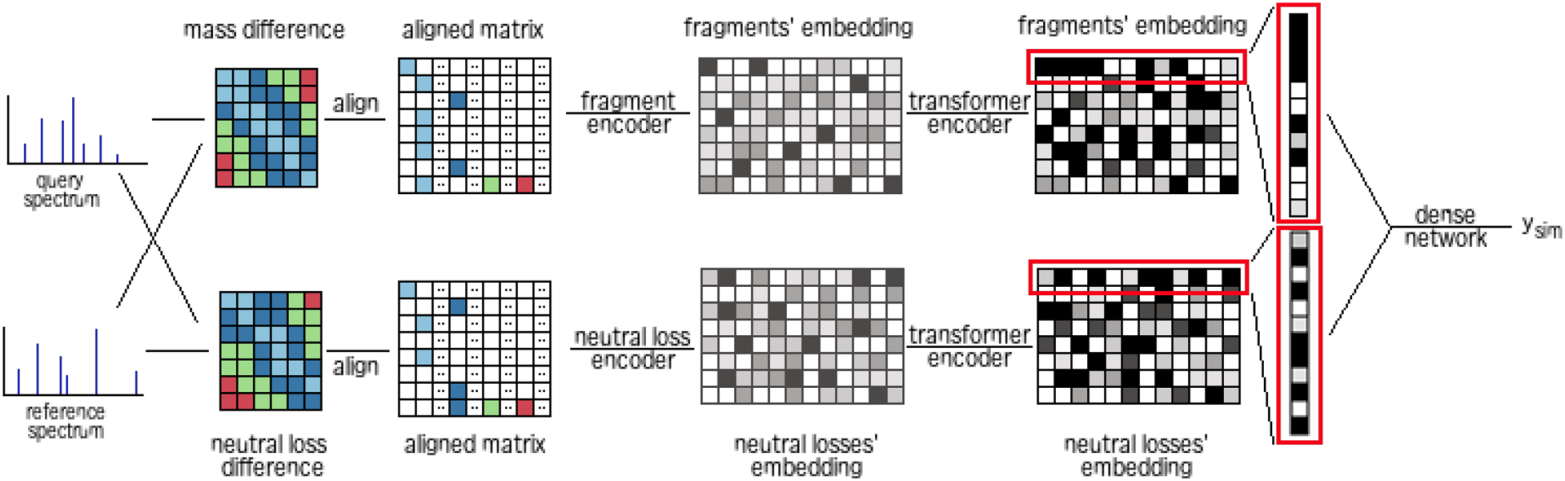
An overview of TransExION model: given a query MS/MS spectrum and a reference MS/MS spectrum as input, we compute the mass difference and neutral loss difference matrices. We then align the rows of these matrices according to their nominal mass, and forward these into a deep neural network. The network encodes the these matrices into feature vectors (or embeddings) and joins them at the end to estimate the similarity of two spectra, $y_{sim}$

Figure 2 illustrates the network workflow. For model training, we assign one MS/MS spectrum of the training pair as “query” and another one as “reference”. Given a query spectrum ($s_q$ fragments) and a reference spectrum ($s_r$ fragments), the model first computes their mass difference matrix (MDM). This matrix consists of $s_q$ rows and $s_r$ columns, and the values indicate the absolute mass difference between query and reference fragments. Structural similarity can be revealed by fragmentation patterns observed in the MDM. In the case of a minor substructure difference (e.g., one chemical moiety extra in the query compound), the substructures of product ions in $s_q$ can either match exactly with $s_r$, leading to zeros in the MDM, or contain the mass difference for that additional chemical moiety. In the latter case, one particular value (e.g., 16 Da for oxygen) can be observed at least once in the MDM, indicating a minor substructure modification.

**Fig. 3:**
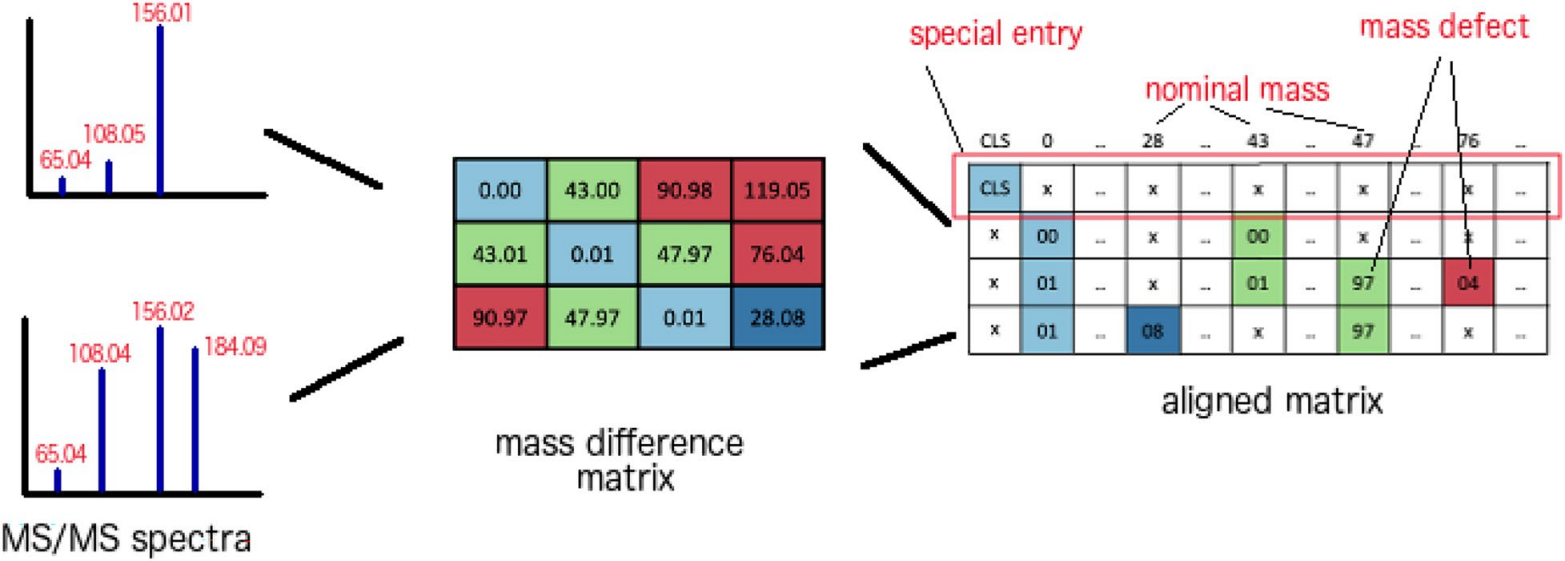
An example of transformation from matrix difference matrix to an aligned matrix

The MDM is then transformed into an aligned matrix that also gathers, for each query fragment, all observed mass differences. At first, the mass difference values are split into the integral and fraction parts, referred to as nominal mass and mass defect, respectively. This new matrix has *n* rows and *m* columns. The number of rows corresponds to the size of the query spectrum ($n = s_q$), while the columns represent nominal masses of mass difference values (${0, 1, 2,\ldots ,m-1}$). We fix here a cut-off of $m = 300$ to consider only the mass differences below 300 Da. The choice of this cut-off is motivated by a focus on minor substructure modifications and the need to control the computational cost. The matrix is then filled with rounded mass defects 0–99 (rounded to two decimal places then multiplied by one hundred). If a nominal mass does not appear in the MDM, that gap is filled with a “pad” value. By doing this, the query fragments are aligned with each other according to the nominal mass, which facilitates the model in detection of the recurrence of a mass difference value. Furthermore, a special entry named *CLS* is added at the beginning of the aligned matrix. It is used to accumulate all similarity information from all the query fragments though the self-attention mechanism in the Transformer. Figure 3 demonstrates the transformation of the mass difference matrix into the aligned matrix.

In parallel, each spectrum of the training pair is converted into a hypothetical neutral loss spectrum (by calculating the difference between the precursor ion and its respective fragment ions). After that, a neutral loss-based MDM and aligned matrix are generated for each pair by repeating the procedures above. In recent studies on spectral library searching, the mirrored neutral loss spectra have demonstrated rich structure similarity information that is complementary to the original spectra [22, 29].

TransExION is composed of two independent Transformer-based networks (one for original and another for neutral loss spectrum) and a fully connected network. The Transformer-based network generates an embedding (a single vector) from input aligned matrix. Two vectors from original and neutral loss spectra are concatenated, which is followed by the fully connected network to predict the final output: a numeric value which indicates the spectral similarity.

Two Transformer-based networks follow the same architecture: a row encoder layer followed by a transformer encoder. For the row encoder, the same transformation is applied to each row of the aligned matrix, resulting in a single feature vector that contains all information about that row. In the case of original spectrum, the mass differences between the query fragment and every reference fragment are encoded. The row encoder for neutral loss encodes the same information for neutral loss.

The output of the row encoders then become the input of the transformer encoder. Thanks to the self-attention mechanism in Transformer architecture, each row vector is able to attend to all row vectors, including itself. In other words, each fragment (or neutral loss) can interact with all fragments (or neutral losses) in the same query spectrum to collect information. The Transformer architecture allows the model to put more emphasis on product ion (or neutral loss) matches and recurring mass differences between multiple query-reference product ions (or neutral losses).

**Fig. 4:**
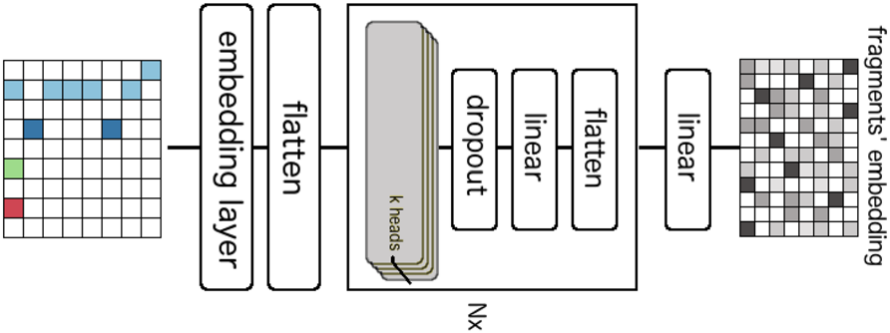
The architecture of a fragment/neutral loss encoder

Figure 4 presents the architecture of a row encoder. It is composed of an embedding layer for mass defects, a flatten operator, a stack of *N* blocks and a fully connected layer. The embedding layer maps each mass defect into a single vector, generating a 3*D* matrix from the aligned matrix. We implemented the embedding for mass defect values of mass differences because of their underlying structural information. The flatten operator then reshapes the 3*D* matrix into a 2*D* one with each row still representing a query fragment or a neutral loss. By doing so, the information of original mass differences is reconstituted through the integration of nominal masses and the embedded mass defects. Next, the stack of *N* blocks project each row in the 2*D* matrix separately and identically to a single vector of *d* dimensions. Each block is composed of a dropout, a fully connected layer and a flatten operator. Before a block is applied, each row is split into *k* parts with same length, named as heads. The dropout and the fully connected layer are applied to these heads, learning local features within the heads. The flatten operator is used to reshape the heads back to rows. After the stack of *N* blocks, the fully connected layer is applied to generate the row embedding. The transformer encoder has the same architecture proposed by Vaswani et al. [30]. All the settings and hyper-parameters of TransExION are summarized in Table 1. At the end, the embedding of the special entry *CLS* is retained as the output.

**Table 1:** Hyper-parameters and their values were used in TransExION model

Component: fragment and neutral loss encoders	
Dimension of embedding layer	32
Number of blocks	2
Number of heads	100 and 20
Hidden dimension	128
Dropout	0.1

Component: transformer encoders	
Number of encoder layers	2
Number of attention heads	4
FFN inner hidden size	256
Hidden dimension	128
Dropout	0.1

Others	
Learning rate	1e$-$4
Weight decay	0.0
Batch size	64

The TransExION model is supplemented with a post hoc analysis of explainability. The goal is to estimate the pairwise relevance between query and reference product ions based on the contribution of each pair to the model output (spectral similarity). This approach was inspired by the Layer-wise Relevance Propagation (LRP) method proposed by Chefer et al. [31]. Basically, we propagated the relevance and gradients corresponding to the pairs that are predicted “highly similar” in structure from layer to layer following the generic Deep Taylor Decomposition. The output of model explainability after spectral similarity estimation is a heat map which reveals, for each query fragment, the most *k* relevant product ions in the reference spectrum.

### Data preparation

Experiments were performed on two data sets that were derived from GNPS [1] and included only positive ion mode spectra. One data set consisted of 11,000 MS/MS spectra of unique compounds. To obtain this data set, “duplicated” spectra of the same molecular structure were merged, by recognition of the identical first layer of InChIKey. Parallel experiments were run with another GNPS-derived data set as recommended by Huber B. et al. [27]. This data set contains 107,734 MS/MS spectra associated with 15,062 different molecular structures (“duplicated” spectra unmerged). For simplicity, we named the two data sets as **mergedGNPS** and **GNPS**, respectively. Spectra from both data sets were randomly split into testing ($n=500$), validation ($n=500$) and training sets (the remaining spectra) according to their underlying structures. Validation sets were used to fine-tune the key hyper-parameters of the model, including the learning rate and the weight decay. Our random data splitting procedure prevents close structural and spectral analogs between testing and training/validation thus information leakage. We observed that very few training data contain structural and spectral information that were both identical to testing data (Fig. A1).

All spectra in this study were pre-processed as follows: the *m*/*z* values of the fragments were rounded to two decimal places, and peak intensities were replaced by relative abundance ($\%$) in relation to the highest peak. All fragments with intensities less than 0.1% or with *m*/*z* less than 10 Da or larger than 1000 Da were removed. We observed, in some training spectra, regions crowded with lower intensity peaks surrounding a large peak. These minor peaks are probably isotope peaks or chemical noise that can lengthen the input spectrum and affect the model output. Therefore, input spectra were denoised by keeping only the highest peak within a 3-Da sliding window.

During the training phase, all training spectra are paired and their structural similarity score is computed. The Tanimoto score on Daylight fingerprints [32] is used to compute the structural similarity. However, proceeding with all spectrum pairs is problematic since the training set becomes enormous and heavily imbalanced towards low Tanimoto similarity scores. To tackle this problem, a procedure is used to generate a much smaller and better balanced set. Basically, we defined an equal width binning, dividing the structural similarity values into $B=10$ equal width bins between 0.0 and 1.0. In each training epoch, each spectrum was scanned over these bins and paired randomly with a different spectrum in each bin.

For model evaluation, we matched every spectrum in both independent test sets with all spectra in their corresponding training sets (**mergedGNPS** or **GNPS**) to mimic spectral library search. To create a balanced and fair-sized testing set, we also applied the equal width binning on structure similarity, choosing randomly utmost $k=3$ pairs in each bin for each testing spectrum. This procedure enables unbiased model evaluation through homogeneous sampling of lower and higher structure similarity query-reference spectrum pairs. It generated 11,425 and 98,625 spectrum pairs as testing data in the **mergedGNPS** and **GNPS** data, respectively.

## Results

### TransExION identifies correct structure analogues

**Fig. 5:**
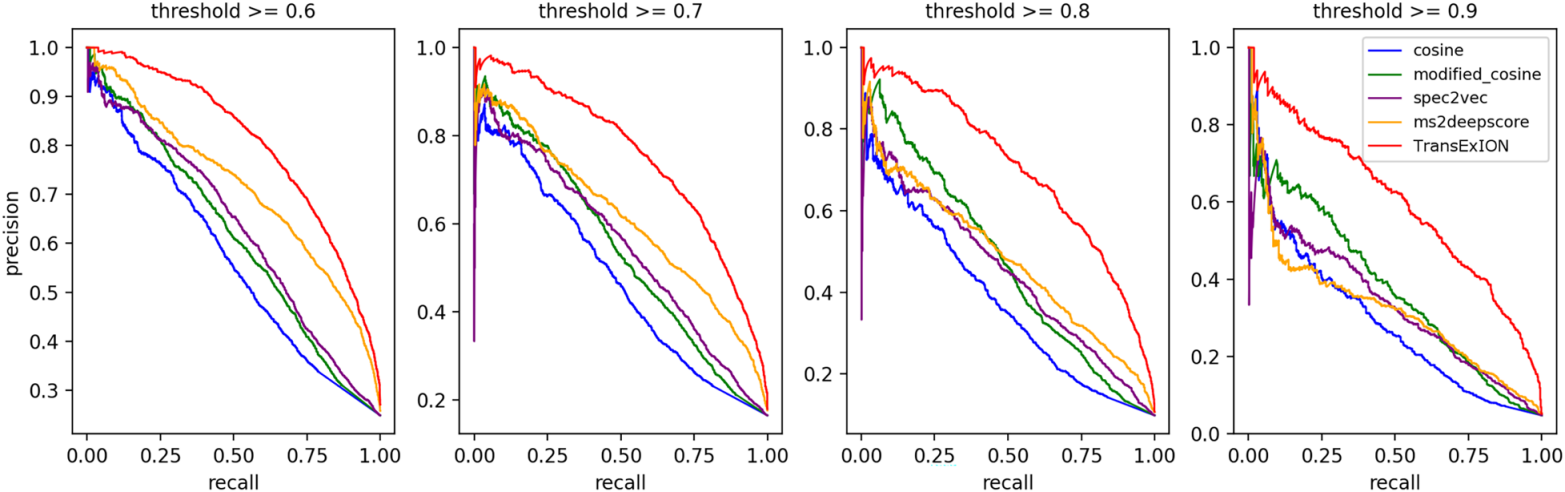
Precision recall curves of several methods to predict high structural similarity between pairs of spectra in the **mergedGNPS** testing data. High structural similarity is defined using four different cut-offs for Tanimoto score, ranging from $>\,0.6$ to $>\,0.9$. The curves illustrate the trade-off between higher precision and higher recall by varying the spectral similarity threshold. TransExION provides a better overall precision/recall combination in the **mergedGNPS** dataset

**Fig. 6:**
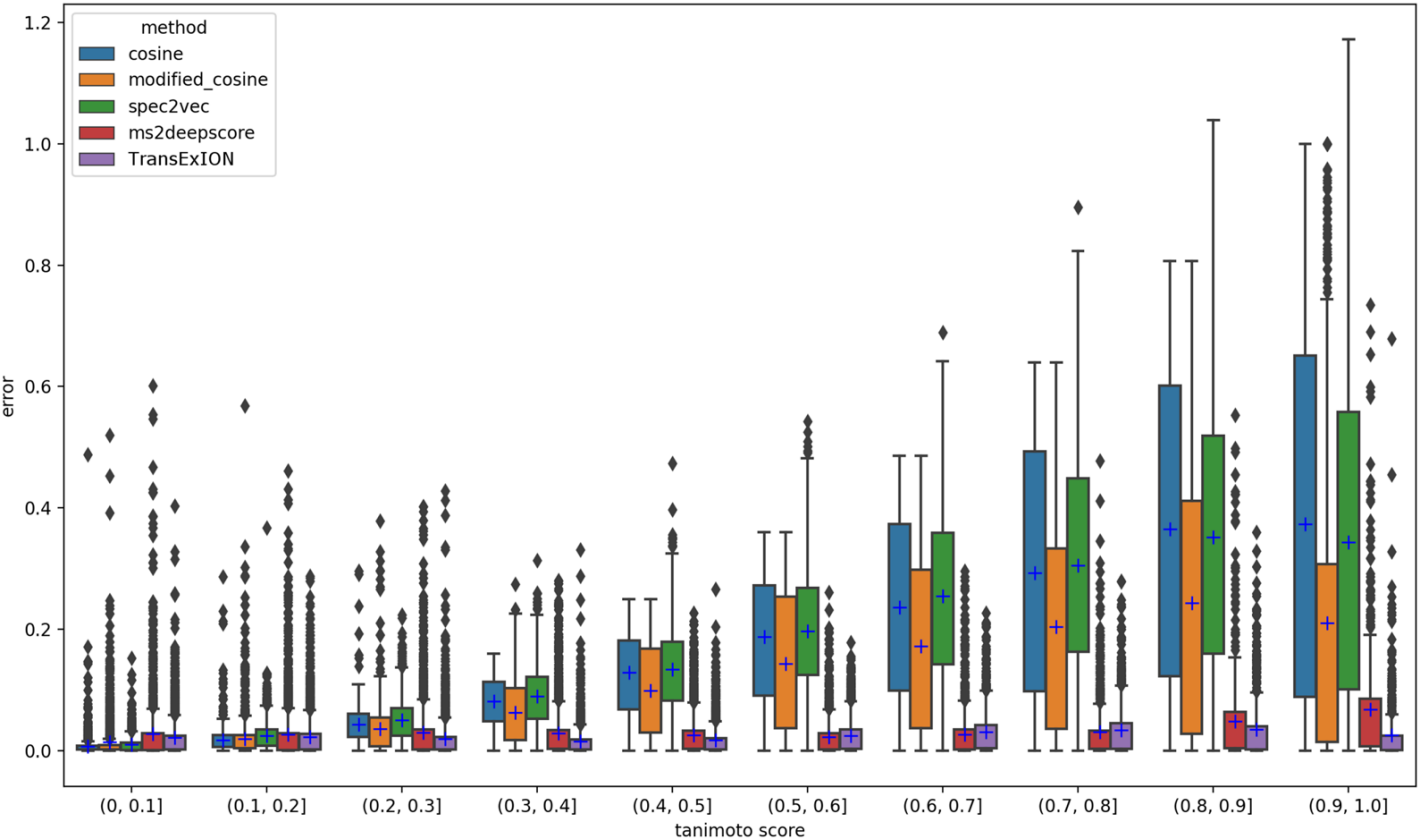
Squared error distribution of different methods on 10 equal width bins of Tanimoto score on **mergedGNPS** dataset. The blue markers indicate MSE values. The prediction error of TransExION is low with minimal variation on different bins while Cosine, Modified Cosine and Spec2Vec increase with higher Tanimoto score and show higher variability

**Fig. 7:**
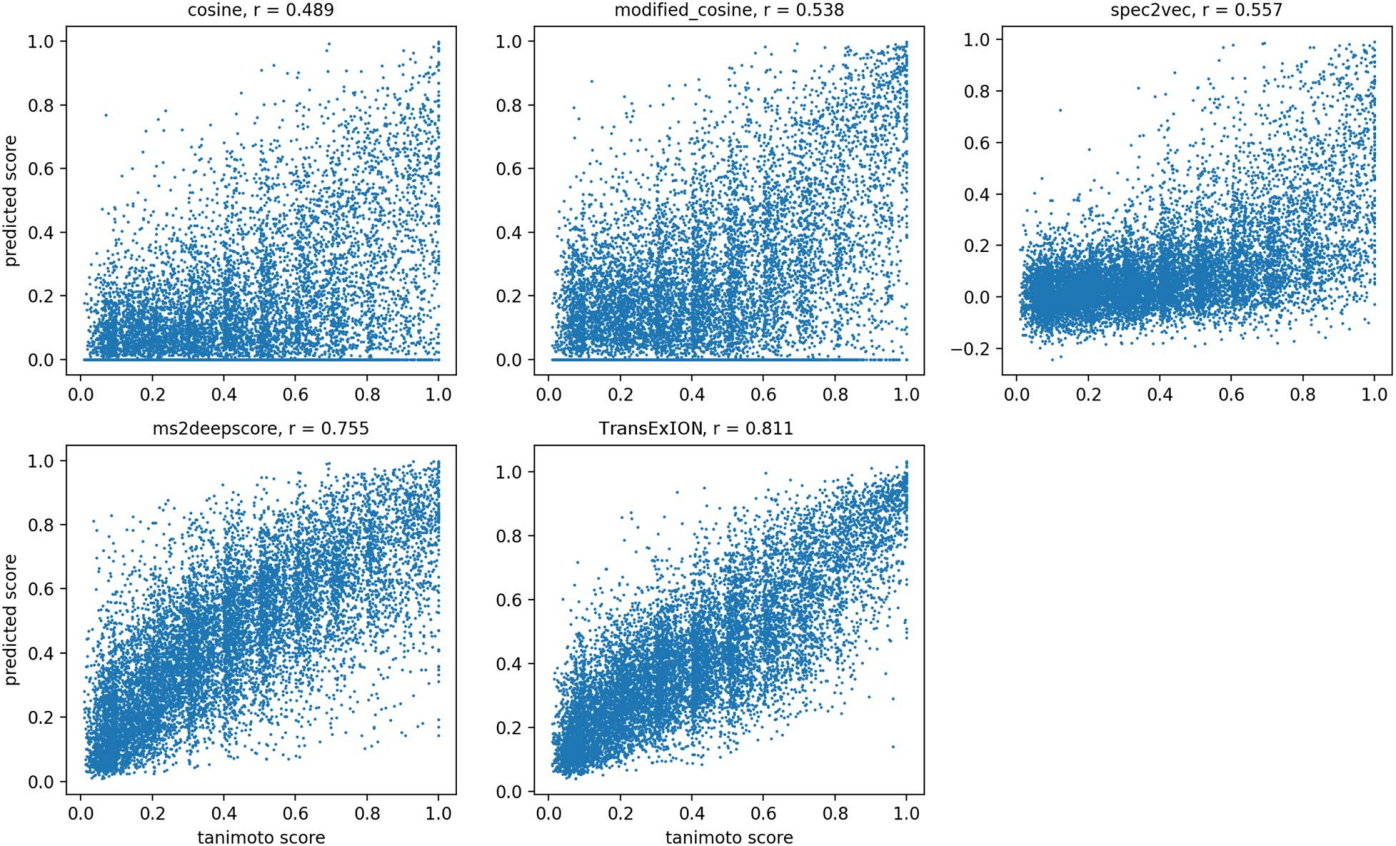
The relationship between the spectral similarity predicted by different methods and the structural similarity measured by Tanimoto score on **mergedGNPS** testing data

To mimic spectral library search and for unbiased evaluation of our model, each spectrum from independent test sets (**mergedGNPS** or **GNPS**) was paired with a selected subset of reference spectra used for TransExION training (“Data preparation” section). Spectral similarity was predicted by TransExION, and the same pairs of spectra were evaluated by popular spectral similarity metrics, namely Cosine [33] and Modified Cosine [34]), along with state-of-the-art models, including Spec2Vec [25] and MS2DeepScore [27]. The experimental results obtained for **mergedGNPS** are displayed in Figs. 5, 6, and 7 while the data for **GNPS** can be retrieved in the supplemental information.

The precision-recall curves in Fig. 5 compare the ability of our model to retrieve structural analogues from spectrum pairs against other similarity metrics in **mergedGNPS** data. It is generally agreed that two compounds are chemically-related analogues if their Tanimoto score is higher than a fixed threshold [27]. To enable rigorous comparison, we applied four different Tanimoto similarity cut-offs ranging from 0.6 to 0.9. TransExION achieves a notably better precision/recall combination than other similarity measures, making itself as an attractive similarity measure for identification of structural analogues in large spectral libraries (10,000 reference spectra used here for evaluation). Moreover, by applying a higher structure similarity threshold, TransExION still maintained a high level of precision/recall. This means that TransExION retrieves structure analogues displaying minor modifications (i.e., very high Tanimoto scores) in an accurate and comprehensive manner, if such analogues are present in the spectral library.

### TransExION prediction aligns with Tanimoto scores

Compared to other algorithms, the shape of TransExION precision-recall curves remains nearly unchanged when different cut-off values are applied (Fig. 5). We hypothesized that the consistency of the curves could be due to good alignment between TransExION spectral similarity score and Tanimoto structure similarity. To evaluate this hypothesis, we applied the same equal width binning for structure similarity (“Data preparation” section), then computed the squared error (SE), which is the distance between predicted score and Tanimoto score) for each query-reference spectrum pair. The box-plots in Fig. 6 reveal the SE distribution of different methods in every structure similarity bin. The blue markers on the box plots indicate the mean squared error (MSE) values. In general, the prediction error of TransExION remained low with minimal variation on different bins for **mergedGNPS** data. Meanwhile, Cosine, Modified Cosine and Spec2Vec increased with higher Tanimoto score and showed higher variability, especially on the bins representing highly similar structures. In fact, the performance of these measures appeared highly unstable when Tanimoto scores were greater than 0.5. Moreover, our method was comparable to MS2DeepScore in most bins in terms of overall prediction error and stability.

Using all spectrum pairs in the **mergedGNPS** testing data, Fig. 7 presents the spectral similarity predicted by different methods against the ground-truth—structural similarity measured by Tanimoto score. Meanwhile, the Pearson correlation coefficient *r* was calculated between the ground-truth and the prediction for each method. Based on the overall shape of point cloud and *r*, we conclude a weak correlation between structural similarity and Cosine/Modified cosine score ($r=0.489$ and 0.538, respectively). Both measures rely on comparing intensities of matching peaks (plus neutral losses for Modified cosine), and their values are spread out between 0 and 1 except when there is a clear structure difference (e.g., Tanimoto distance $<\, 0.2$). With a stronger overall correlation ($r = 0.557$), Spec2Vec displays a quite homogeneous distribution between ${-}$ 0.2 and 0.2 for spectrum pairs with lower Tanimoto scores (0–0.6). However, the prediction is spread out in the 0.6–1 Tanimoto score range, probably because Spec2Vec, as an unsupervised method, explores relationships between product ions without using structural information. In contrary, MS2DeepScore and TransExION are both supervised models trained with structural similarity as ground truth, hence they both reveal a strong correlation with the ground-truth ($r = 0.755$ and $r = 0.811$, respectively). Although a perfect correlation was not achieved, both models are highly reliable in separating high (e.g., Tanimoto distance 0.6–1), mid (0.4–0.6), and low structural similarity pairs ($<\, 0.2$). The slight out-performance of TransExION (higher correlation coefficient *r*, less widespread point cloud) can be linked to the explicit encoding of product ion mass differences into the model. These mass differences can also facilitate the post hoc explanation for the model’s outcome, which is presented in the next section.

### TransExION allows model explainability

**Fig. 8:**
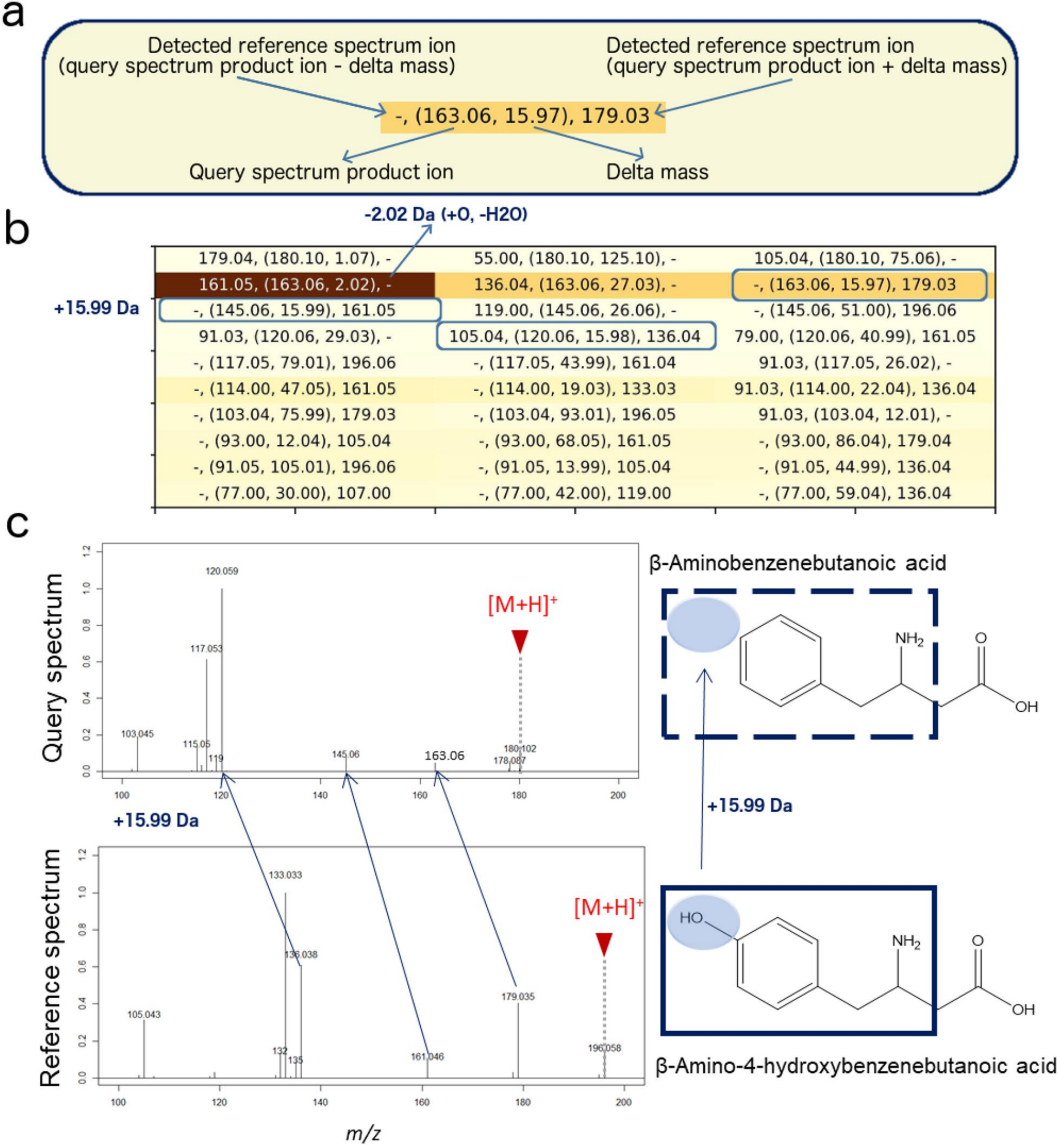
First example of model explainability. **a** Annotation of the values in the heatmap. **b** Heatmap generated by model explanability analysis.The darker the cell is, the more important it contributes to the similarity score. **c** Visualization of query and reference structures by highlighting the modified substructure

**Fig. 9:**
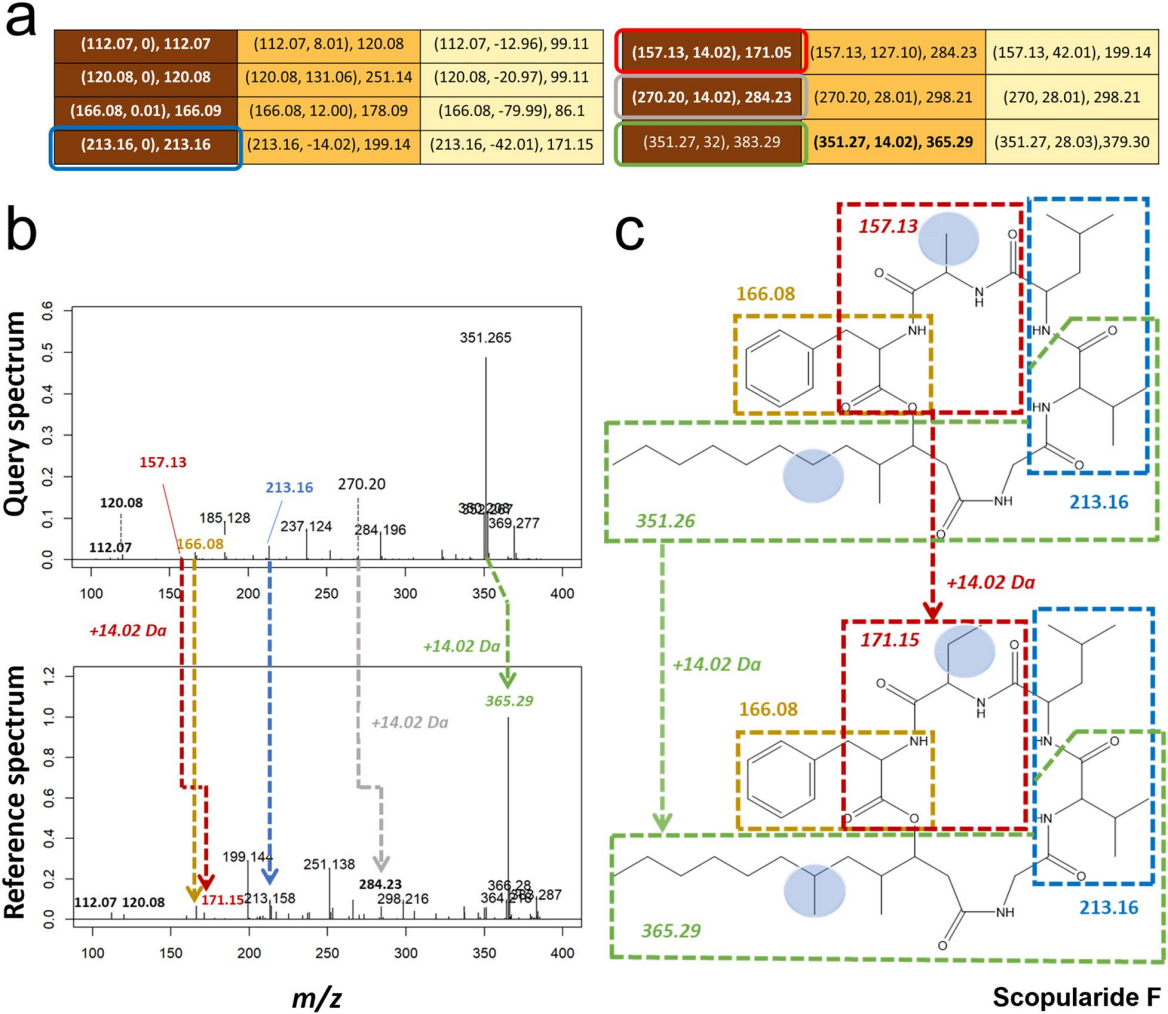
Second example of model explainability. We display here filtered **a** heatmaps that only contain query-reference product ion pairs used for structure elucidation: exact matches or with a 14 Da offset. These associations are highlighted in **b** by the arrows between query (upper plot) and reference spectrum (lower plot). In **c**, while the exact matches ($$ m/z = 166.08$$ and 213.16) confirm the amino acid composition of the query spectrum, the two 14 Da offsets indicate methylene loss on the 171.15 and 365.281 substructures

In this section, model explainability analysis, which was described in “Model architecture” section, was performed on the testing data of **mergedGNPS**. For each query spectrum, we first extracted the top $q = 20$ product ions based on their overall contribution to the structure analog prediction. Next, each product ion was evaluated against the top $k = 3$ most associated product ions in the corresponding reference spectrum. The relevance matrix was then visualized in a heatmap as the output of model explainability, and is explained in Fig. 8a. Two examples displayed in Figs. 8 and 9 demonstrate the structural relatedness of highly associated query and reference product ions revealed by the relevance matrix.

In the first example, TransExION similarity between DL-beta-Homophenylalanine (Spectrum ID: splash10-00xr-0900000000-e86eefea78d902b2e731) and its best matched reference spectrum (splash10-001r-1900000000-f5b35a51eb71bdd8a479) was 0.74. With a Tanimoto similarity of 0.83, the matched reference is a clear structure analog of DL-beta-Homophenylalanine as it corresponds to the para-hydroxylated form of this amino acid. Although the two compounds only differ by one hydroxyl group on the aromatic ring, Cosine and Spec2Vec similarity fell below 0.15 likely due to the lack of common product ions. Meanwhile, Modified Cosine was able to detect structural similarity correctly, 0.84 thanks to shared neutral losses. TransExION was also able to predict the overall structural similarity correctly and provided the relevance matrix to explain the similarity via the different product ion pairs (Fig. 8b). A clear shift of $+15.99 Da$ is observed between several product ions in the query and reference spectra which originated from the difference of one oxygen in the elemental composition in the reference structure ($m/z=163.06 \rightarrow m/z=179.03, m/z=145.06 \rightarrow m/z=161.05$ and $m/z=120.06 \rightarrow m/z=136.04$), displayed in Fig. 8c. These $+15.99 Da$ shifts are also evident in the relevance matrix for this match (Fig. 8b). Moreover, the transition with highest relevance in the matrix was a ${-}$ 2.01 Da shift ($m/z=163.06 \rightarrow m/z=161.05$) which can be readily explained by the difference of one additional oxygen ($+15.99 Da$) between DL-beta-Homophenylalanine (query) and its hydroxylated counterpart (reference), combined with a neutral loss of $\hbox {H}_2\hbox {O}$ from the hydroxybenzyl moiety (${-}$ 18.01 Da) in a fragmentation reaction similar to that explained in Chai et al. [35].

In the second example, the query spectrum of a cyclic lipodepsipeptide (Spectrum ID: splash10-0udi-2014761900-e58065981f99435865cd) was correctly matched with the reference spectrum of its analog Scopularide F (splash10-00vi-5126930200-7b2b1b2440dcd21ad598), differing in two functional groups. Despite the presence of many product ions in both spectra, the number of shared product ions or neutral losses was relatively low, and no other metrics scored the spectral similarity high enough to reflect their structural similarity. Cosine, Modified Cosine, Spec2Vec, and MS2DeepScore estimated the similarity 0.29, 0.54, 0.74, and 0.74, respectively. Again, TransExION assigned a high similarity score of 0.92 for the spectrum pair. As apparent from a selection of the relevance matrix (Fig. 9a, the full relevance matrix can be found in Supplementary Fig. C5), TransExION prediction was predominantly explained by the identical matches of several peaks. Interpretation of the reference spectrum identified these product ions as related to amino acid residues such as Leucine ($m/z=112.07$), Phenylalanine ($m/z=120.08, m/z=166.08$) or dipeptide fragments (Val-Leu, $m/z=213.16$). Furthermore, the TransExION relevance matrix assigned several pairs of associated query-reference product ions with a ${-}$ 14.02 Da offset (Fig. 9b), suggesting a difference of a methylene group ($\hbox {CH}_2$) from Scopularide F. Since the two molecules differ by 28.03 Da, we suspected that the query compound corresponds to the loss of methylene groups at two distinct locations. The product ions observed in the query reference spectrum (Fig. 9b) and displayed in the relevance matrix, helped to annotate the query structure (Fig. 9c): the $m/z=157.13 \rightarrow m/z=171.15$ transition (red) indicated a valine instead of 2-aminobutyric acid residue, while the $m/z=351.26 \rightarrow m/z=365.29$ transition (green) (in combination with the unchanged Val-Leu product ion at $m/z=213.16$, blue) localized the additional methyl on the lipid moiety. This is further confirmed by the $m/z=270.20 \rightarrow m/z=284.23$ transition (grey) that does not include the valine residue.

Both examples clearly show how the explainability obtained through the relevance matrix assists in the MS-based structure elucidation of analogous compounds and helps to pinpoint both the chemical nature and sites of modification of unknowns, compared to their spectral library match.

### Molecular networking using TransExION as similarity metric

**Fig. 10:**
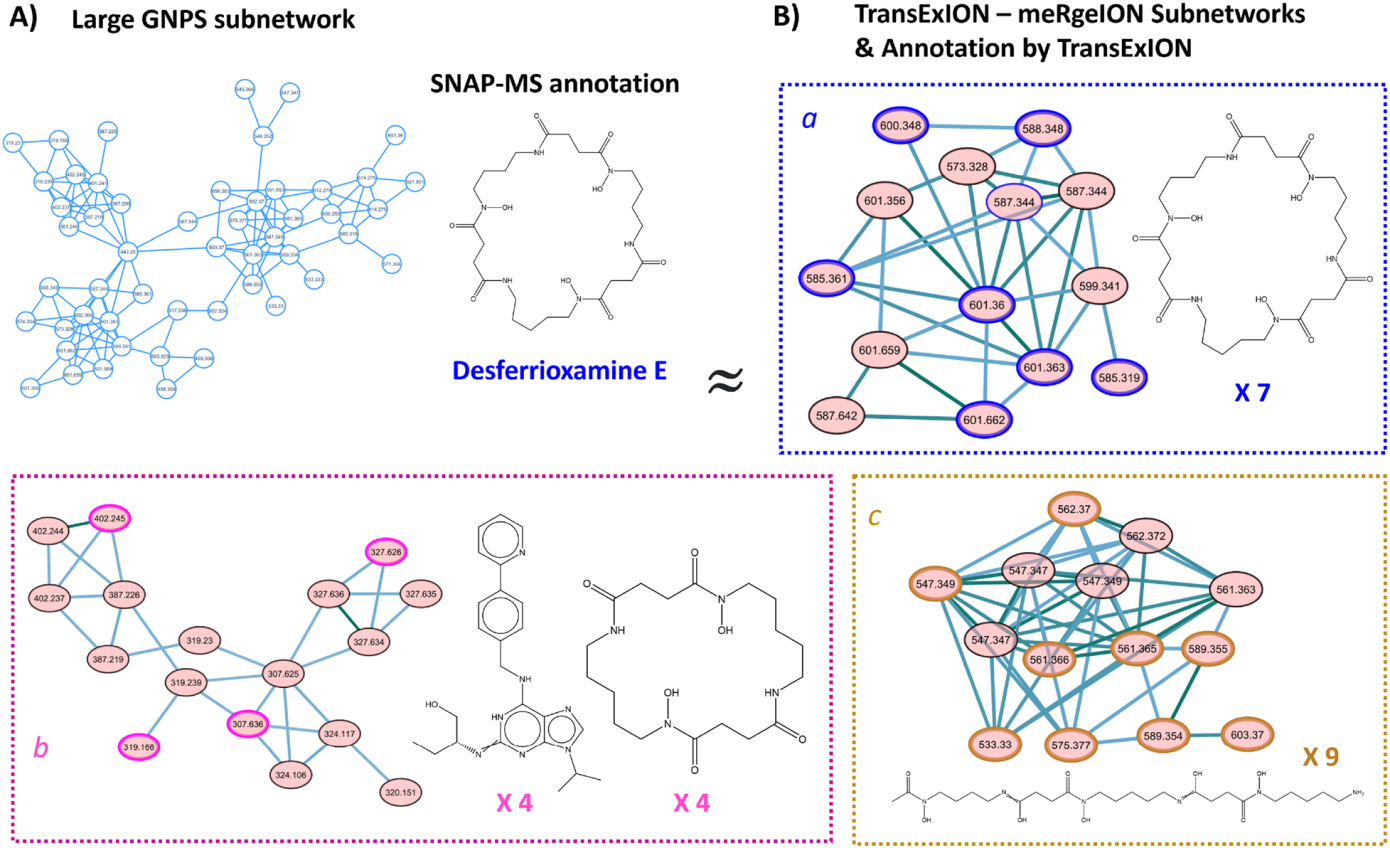
Using TransExION as an alternative spectral similarity metrics for molecular networking: **A** A large sub-network in the GNPS molecular network of marine bacteria extract, annotated as Desferrioxamine-related analogs by SNAP-MS, **B** three sub-networks found by TransExION-meRgeION showed a strong node overlap with the GNPS sub-network. Analogue search by TransExION linked each sub-network to a different compound family based on the most frequent annotations

Since TransExION spectral similarity strongly aligns with the structural similarity ground-truth (correlation coefficient $r = 0.811$), it is powerful in separating high, mid and low structural similarity pairs. Such property suggests the potential of our method in separating experimental spectra of complex chemical mixtures) into clusters representing different compound classes, which is frequently achieved by GNPS molecular networks built from heuristic similarity measures such as Modified Cosine [1]. Recently, alternative algorithms such as SNAP-MS were developed for improved molecular networking (MN) [36]. To assess the potential of TransExION as an alternative spectral similarity measure to be used in MN, we reproduced a SNAP-MS derived molecular network obtained from a 925-sample, marine bacteria extract library [36]. The pairwise spectral similarity was computed in TransExION for 2161 spectra and the obtained values were imported into meRgeION for molecular networking [37]. Edges were filtered to have a TransExION score above 0.6 without the requirement for minimum matched peaks (arbitrarily set at 6 in many GNPS networks). All other network parameters, such as *topK* and the maximum size of a molecular family, were kept the same as in the original manuscript. In addition, all experimental spectra (nodes) were annotated through analogue search against the mergedGNPS spectral library (by computing the TransExION spectral similarity against all reference spectra). We kept the top three structure analogues for each node if the similarity score was above 0.6. Encouragingly, the molecular network, using TransExION as similarity metric, captured all seven sub-networks that were assigned confidently with compound families by SNAP-MS in the original paper (Figure *D*7). However, a difference in network topology for some clusters was detected. For instance, a large 52-node component in the GNPS network, was divided into three smaller clusters of 14(a), 17(b) and 14(c) nodes, respectively in the TransExION-meRgeION network (see Fig. 10). This division potentially indicates the presence of sub-families within the same network component. Interestingly, half of the nodes in cluster were annotated as Desferrioxamine by TransExION, with other top hits obtained for the clusters *b* and *c*. So, while the entire MN component was proposed as Desferrioxamine-related by SNAP-MS, TransExION assigned the same compound family to a smaller set of spectra. As such, the current example shows that by embedding TransExION into MN workflows, meaningful networks can be obtained. The difference in network topology and analog search results could provide an alternative explanation of existing MS/MS data.

## Conclusions

Identification of small molecule structure from MS/MS spectra plays a crucial role in modern life sciences and bio-analytical research. In this study, we tackled a difficulty of using spectral library search to assign structure analogues to unknowns and developed a deep learning based method to confidently predict spectral similarity via structure similarity approximation. Our approach explores the latent links between mass differences of product ions and structural relatedness, which elegantly covers minor substructure modifications in addition to exact substructure matches. Furthermore, previous studies demonstrated that metabolites often share substructures, resulting in similar patterns in their MS/MS spectra [37, 38]. The attention mechanism in our transformer-based networks enables the detection of both co-occurring product ions (or neutral losses) and recurring substructure modifications.

We first evaluated how accurately TransExION retrieves structure analogs from a large spectral library using two independent test sets. Experimental results show that TransExION outperforms simple, heuristic similarity measures, such as Cosine and Modified Cosine, and the unsupervised Spec2Vec model. It also outperforms the deep-learning model MS2DeepScore in both test sets, especially when nearly-identical structure matching is desired (high Tanimoto distance cut-off). Another advantage of TransExION is its overall low prediction error and high stability throughout the entire structure similarity range. Based on that, we can imagine using TransExION as a spectral similarity alternative in complex mixture analysis. Using the pair-wise spectral similarities computed by TransExION, we built a molecular network of bacteria extracts and retrieved sub-networks representing confidently-identified chemical families[36].

Compared to other deep learning models for structure elucidation, the TransExION framework is built with a unique post hoc explanation module. The explanation of query product ions is achieved based on the contribution of query-reference product ion associations to spectral similarity prediction. According to experts’ evaluation, most of the important product ion pairs found by TransExION correctly reflect the chemical relatedness, that is, either an exact substructure match or a minor modification. Moreover, the TransExION model can explain simultaneous modifications of distinct functional groups from the reference compound. In practice, the post hoc explanation module can provide a good starting point of the structure elucidation of unknowns by linking query with reference product ions, since the substructures of the latter can be easily assigned by analyzing the fragmentation mechanism of the reference compound.

Next to spectral library search, structure database search is another popular method in identifying small molecule structure from MS/MS spectra. Although structure databases usually contain a much larger fraction of compounds than current spectral libraries, both approaches are intrinsically restricted to compounds in the database. Recently, a database-free approach emerged in which the structures are generated directly from MS/MS spectra via deep generative models [5, 21]. For all approaches above, finding good spectra embedding is the key to performance improvement of machine or deep learning models. In this regard, the intermediate spectra representation generated by TransExION can be used seamlessly by other models to further enhance unknown structure elucidation.

### Supplementary Information

Supplementary Material 1

## Data Availability

A PyTorch implementation of TransExION can be found at Github https://github.com/banhdzui/TransExION.git. The fully trained model on mergedGNPS data and the data can be downloaded from https://zenodo.org/record/8175528. The GNPS data which has been created by the authors of MS2DeepScore [27] can be downloaded from https://zenodo.org/record/4699356.

## Declarations

### Competing of interests

There is no competing of interests.
