# Supplementary material for: TransExION: a transformer based explainable similarity metric for comparing IONS in tandem mass spectrometry
Source: J Cheminform. 2024 May 28;16:61. doi: 10.1186/s13321-024-00858-5 (PMC11134763; doi:10.1186/s13321-024-00858-5)
Supplement: Supplementary file 1 — Supplementary Material 1 [file 13321_2024_858_MOESM1_ESM.pdf]

TransExION: A Transformer - based Explainable similarity metric for comparing IONS in Tandem Mass Spectrometry

Appendix A Data leakage

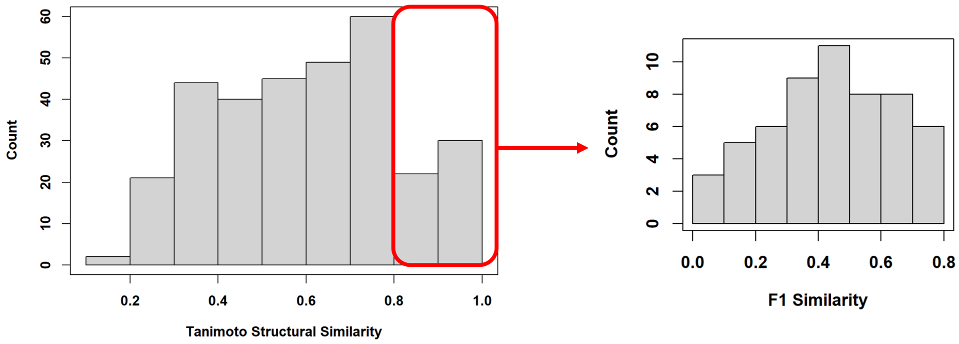

Fig. A1 Evaluation of data leakage by random splitting. For each testing structure, we calculated the Tanimoto similarity against its closest structure in the training set. Only 56 out of 500 testing structures are very close to their counterparts in the training set by a Tanimoto similarity > 0.8 (highlighted by the red rectangle). For these 56 pairs, we measured the $F1$ similarity between training and testing spectra. While the highest $F1$ score is 0.8, most pairs have a spectral similarity below 0.5, indicating that very few training data contain structural and spectral information that are both identical to testing data.

Appendix B Experimental result on GNPS dataset

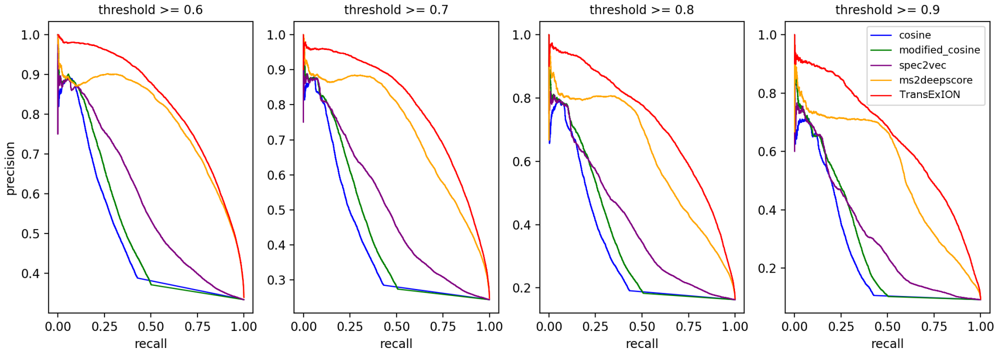

Fig. B2 Precision recall curves of the methods in predicting the high structural similarity between pairs of spectra on **GNPS** testing data. The high structural similarity is defined using four different cut-off for Tanimoto score, ranging from > 0.6 to > 0.9. The curves illustrate the trade-off between higher precision and higher recall by varying the spectral similarity threshold. TransExION provides a better overall precision/recall combination in **GNPS** dataset.

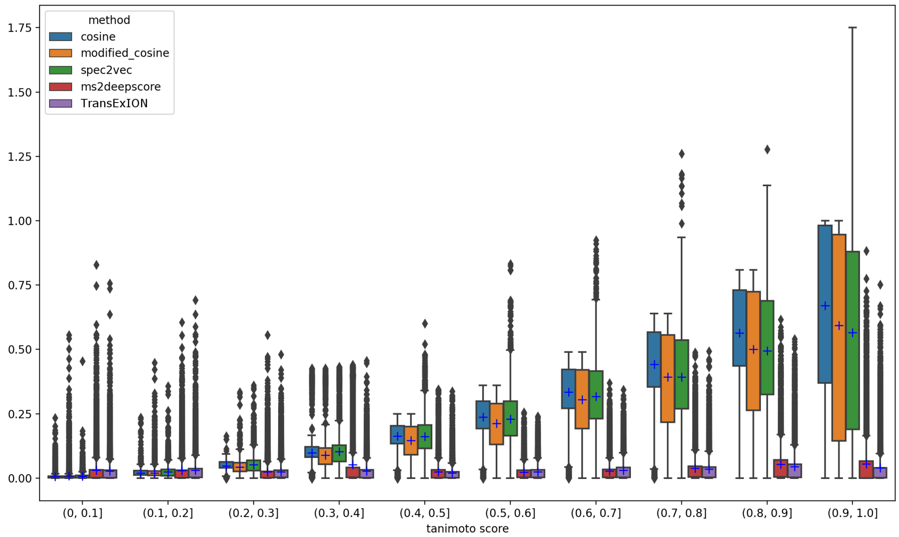

Fig. B3 SE distribution of different methods on 10 equal width bins of Tanimoto score on **GNPS** dataset.

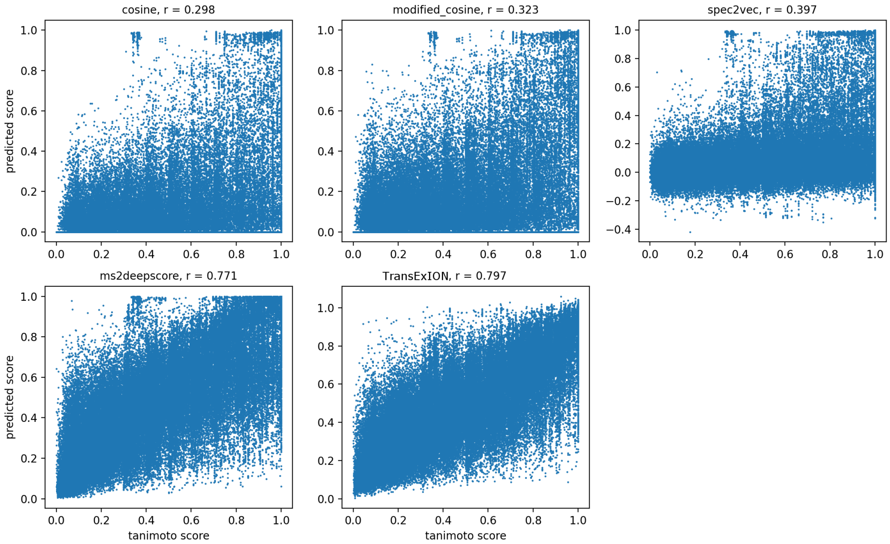

Fig. B4 The relationship between the spectral similarity predicted by different methods and the structural similarity measured by Tanimoto score on **GNPS** testing data.

Appendix C Model explainability

Figure C5 illustrates the heatmap generated for the query spectrum ID=RALTUMFCLLNZRF and the reference spectrum ID=DFFYMZZNXYPOIR. From the heat map, we observed that the estimation was made based on the identical fragments, several occurrence of the mass difference of 0.0 on the first column of the heatmap. The mass difference of 14.02 also occurs repeatedly at the fragments of $86.10, 157.13, 213.16, 284.20, 351.27, 436.35$.

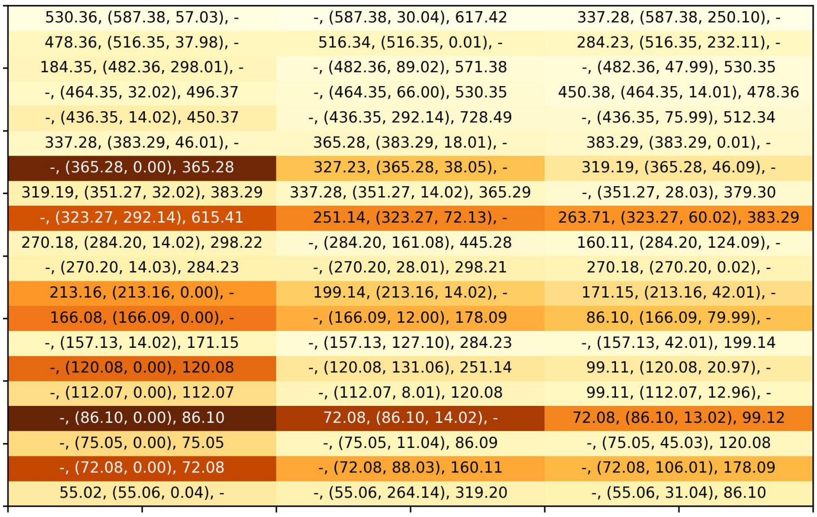

Fig. C5 Visualization of the heatmap of the query spectrum ID=RALTUMFCLLNZRF and the reference spectrum ID=DFFYMZZNXYPOIR.

Figure C6 enumerates the top $K = 50$ most frequent mass difference values that were used to explain for highly similarity in structure of the spectra in **mergedGNPS** testing data. The value of 0.0, which indicates the identical match, is the most common one with more than 7000 occurrences. The value of 14.02 is the second most frequency value, which is associated to the fragmenting of CH_2. Following the value of 14.02 is the value of 0.01, which also indicates the identical match but with a tolerance of 0.01. From the plot, we observed other interesting values, such as 12.00 and 15.99, which are the atomic mass of carbon (C) and oxygen (O).

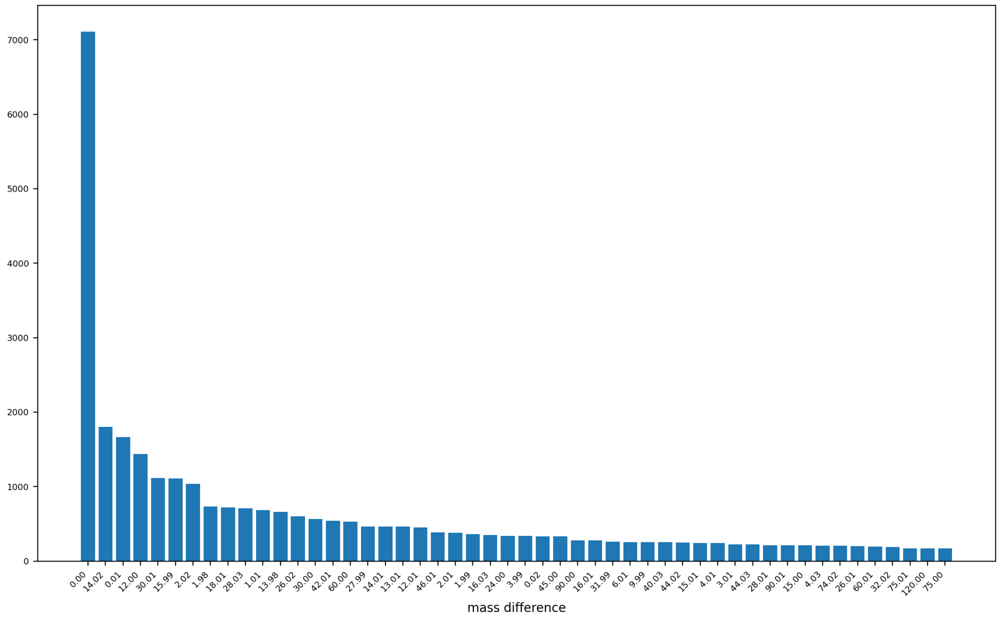

Fig. C6 Top K=50 most frequency mass difference values which were used by the post-hoc explanation model to explain for the MS/MS spectra which were predicted highly similar structure by our model in the mergedGNPS testing data.

Appendix D Molecular networking

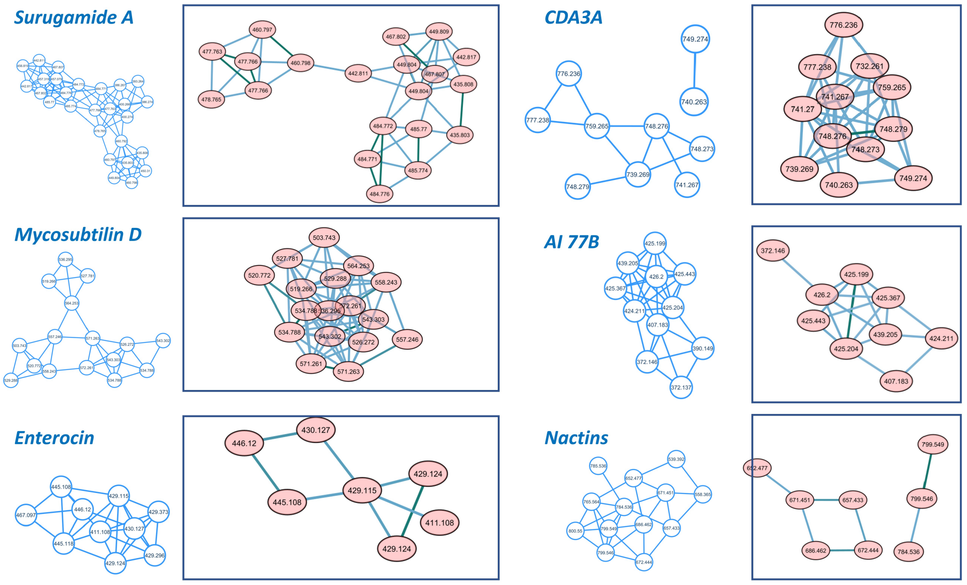

Fig. D7 Six other sub-network found in the TransExION-meRgeION molecular network (right plots) that highly overlapped with SNAP-MS-annotated GNPS sub-networks (left plots).
